# Remote patient monitoring strategies and wearable technology in chronic obstructive pulmonary disease

**DOI:** 10.3389/fmed.2023.1236598

**Published:** 2023-08-17

**Authors:** Felix-Antoine Coutu, Olivia C. Iorio, Bryan A. Ross

**Affiliations:** ^1^Respiratory Epidemiology and Clinical Research Unit, Centre for Outcomes Research and Evaluation, Research Institute of the McGill University Health Centre, Montreal, QC, Canada; ^2^Department of Medicine, McGill University, Montreal, QC, Canada; ^3^Division of Respiratory Medicine, Department of Medicine, McGill University Health Centre, Montreal, QC, Canada; ^4^Montreal Chest Institute, McGill University Health Centre, Montreal, QC, Canada

**Keywords:** chronic obstructive pulmonary disease, exacerbations of COPD, remote patient monitoring, wearable electronic devices, COVID-19 pandemic

## Abstract

Chronic obstructive pulmonary disease (COPD) is highly prevalent and is associated with a heavy burden on patients and health systems alike. Exacerbations of COPD (ECOPDs) are a leading cause of acute hospitalization among all adult chronic diseases. There is currently a paradigm shift in the way that ECOPDs are conceptualized. For the first time, objective physiological parameters are being used to define/classify what an ECOPD is (including heart rate, respiratory rate, and oxygen saturation criteria) and therefore a mechanism to monitor and measure their changes, particularly in an outpatient ambulatory setting, are now of great value. In addition to pre-existing challenges on traditional ‘in-person’ health models such as geography and seasonal (ex. winter) impacts on the ability to deliver in-person visit-based care, the COVID-19 pandemic imposed additional stressors including lockdowns, social distancing, and the closure of pulmonary function labs. These health system stressors, combined with the new conceptualization of ECOPDs, rapid advances in sophistication of hardware and software, and a general openness by stakeholders to embrace this technology, have all influenced the propulsion of remote patient monitoring (RPM) and wearable technology in the modern care of COPD. The present article reviews the use of RPM and wearable technology in COPD. Context on the influences, factors and forces which have helped shape this health system innovation is provided. A focused summary of the literature of RPM in COPD is presented. Finally, the practical and ethical principles which must guide the transition of RPM in COPD into real-world clinical use are reviewed.

## Introduction

We are in an unprecedented period in human history marked by a longer life expectancy and a global aging of the human population. With this remarkable basic sanitary, public health and healthcare-driven success, however, come new pressures and challenges for these same health systems. The accruement in the number and severity of chronic diseases with age has led to multimorbidity and increased complexity of care (1). There is a remarkable increase in the burden of chronic medical conditions (2), which will require innovation and revision to the traditional care model.

*Remote patient monitoring (RPM)* enables the collection of patient health data using peripheral measurement devices or specific questionnaires about their condition without necessitating an in-person visit to obtain these measurements. Typically used in the comfort of the patient’s home environment, this form of monitoring involves the real-time transfer of data to a dedicated platform where healthcare professionals can receive and/or access it. Remote patient monitoring solutions may therefore possess the potential to reduce healthcare costs and increase patient quality of life (3).

## Chronic obstructive pulmonary disease (COPD) and exacerbations of COPD (ECOPDs): a paradigm shift

*Chronic obstructive pulmonary disease (COPD)* is a very common and progressive respiratory condition characterized by chronic breathlessness, a gradual decline in lung function, and reduced quality of life (4). COPD alone was responsible for 3.23 million deaths worldwide in 2019 and has become the third-leading cause of death (5). The global estimated prevalence is 11.7%, and this estimate is projected to increase due to global population aging and due to the growing rates of both smoking and non-smoking exposures in low-and middle-income countries (LMICs) (6). Cigarette smoke (7), occupational exposure to toxic particles, and outdoor and indoor air pollution are all relevant risk factors (6).

The natural history of COPD is characterized by a progressive decline in lung function over time and is also marked by acute episodes of increased symptoms and physiological alterations known as *exacerbations of COPD (ECOPDs)*. While in the acute setting ECOPDs are clinically important events, frequent and severe ECOPDs can also lead to irreversible airway damage and worsening in chronic lung function (8, 9). The overall rate of ECOPDs and COPD hospitalizations continues to increase, partly due to increasing COPD prevalence and more severe forms of disease associated with longer lifespans (6, 10). For example, from 2010 to 2015, the rate of hospitalization for ECOPDs increased from 83 to 86 per 100,000 individuals (10), and COPD remains a top cause of hospitalization amongst all adult chronic diseases. The significant contribution of hospitalizations to the total cost of COPD, amounting to a staggering $50 billion in the United States alone, clearly indicates that COPD poses a substantial burden on healthcare systems (11).

A new ECOPD definition and classification was recently put forth by Celli et al. (12) in the *Rome Proposal*, in part to address issues with the pre-existing framework which had retrospectively classified exacerbations by the way the treating clinician managed the patient rather than using, for example, objective physiological criteria. Using six objectively measured variables in addition to symptom scores, clinicians can use the more ‘objective’ criteria presented in the Rome Proposal to classify ECOPDs as ‘mild’, ‘moderate’ or ‘severe’. These variables include not only dyspnea but also *oxygen saturation (SpO_2_)*, *respiratory rate (RR)* and *heart rate (HR)*, as well as serum C-reactive protein (CRP), and in some cases, arterial blood gas (ABG) values (12). While this will require prospective validation, early enthusiasm and support for this new definition/classification criteria by the international COPD community is reflected by its inclusion in the latest Global Initiative for Chronic Obstructive Lung Disease (GOLD) 2023 Report (6).

## The COVID-19 pandemic

Even predating the reporting of the first observed COVID-19 cases (13) and the global pandemic that followed, there had been notable technological advancements in the development of increasingly precise and compact devices with extended battery life. The COVID-19 pandemic itself then further complicated the delivery of healthcare, including challenging the conventional chronic management of patients with COPD through decreased availability of in-person clinics and recurrent pulmonary function lab testing closures. From a therapeutic perspective, temporary shortages in inhaled medications and the absence of in-person pulmonary rehabilitation programs compounded these difficulties (14). These COPD-specific challenges, combined with challenges which affected all patients with chronic disease including social distancing, isolation, and area-wide lockdowns, led to a recognition of the need to develop new disease monitoring approaches (6, 15). This was embraced also by health systems. In 2019, for example, the Centers for Medicare and Medicaid Services (CMS) established billing codes for remote monitoring (16), and in 2021 the use of remote monitoring platforms in traditional Medicare had increased by six-fold compared to pre-pandemic levels (17).

## Remote patient monitoring

*Remote patient monitoring (RPM)* presents an interesting and innovative approach to address the multiple challenges faced by burdened health systems around the world, both newer issues (pandemic-associated) and more longstanding ones (geographic issues with rural/underserved regions, the ever-growing burden of effective in-person chronic disease management for the aging global population, and so on). Applications in the effective management of COPD span across the ‘chronic’ (regular respiratory monitoring and remote pulmonary rehabilitation delivery) and ‘acute’ (early detection of new exacerbations and ensuring adequate recovery) conditions.

RPM in COPD thus far has encompassed a variety of equipment, platforms, and strategies. Conventional approaches have included remote lung function testing on fixed/stationary devices such as peak flow meters and oscillometers, closely resembling the data collection process employed in hospitals and ambulatory clinics, which typically yields one measurement per day (3). More recent emerging approaches have incorporated ‘wearables’, biometric devices which can be worn for prolonged periods while collecting relevant physiological parameters in daily life (17, 18). These wireless, non-invasive, and self-contained devices can be attached to the human body or to clothing (19). The incorporation of wearables within RPM platforms has facilitated near-continuous remote collection and transmission of physiological data in ambulatory patients, enhancing the richness and resolution of data collected when compared to more traditional approaches and ushering in an era of sophisticated personalized medicine (17, 20–22). A semi-structured approach was followed in order to extract articles from the existing literature (see Supplementary material). The following is a focused summary of RPM in COPD following an independent review and appraisal of each article. A concise summary of the most relevant articles featured in this review can be found in the Supplementary Table 1.

## Discussion

Current ‘medical’, ‘research-grade’ and ‘consumer-facing’ wearable biometric devices are worn on various parts of the human body, including the head, limbs, and torso (23). Wristbands (24, 25), armbands (26), vests (27, 28), upper thorax stamps and bands (29), and rings (30) are all commercially available. The types of physiological data collected can include blood pressure, HR and its variability (HRV), RR and its variability (RRV), SpO_2_, activity, body temperature and metabolic function, sleep metrics and autonomic function including electrodermal activity (31).

There are pros and cons to each type of device as it relates to technical performance (quality/quantity of data collected), patient satisfaction, and ease of overall use. Wristbands, smartwatches, and rings are highly user-friendly, comfortable to wear, and possess versatile functionality which makes them highly suitable for prolonged use (32). These devices largely utilize optically obtained photoplethysmographic (PPG) signals which measure the intensity of light that penetrates through the skin to estimate the frequency and amplitude modulation of the cardiac pulse. Indirect estimations of RR are possible through frequency modulation (FM), by analyzing the variation in pulse frequency (33, 34). While this method offers several advantages, the indirect measurement of RR through the derivation of the PPG waveform may risk limiting precision compared with devices capable of directly measuring thoracic expansion, especially during strenuous movements or during exercises which can induce movement artifact (35).

Wearable vests, shirts, and bands are highly accurate in measuring cardiac and respiratory parameters in patients with COPD given the proximity to the heart to detect electrical activity and the use of chest expansion for detailed respiratory measurement. They are, however, limited by a sensation in some patients of discomfort by being mechanically restrained particularly during inspiration/expansion of the thorax (28). Given the diverse range of devices available and under continuous redevelopment, clinical researchers have a variety of options available to choose from. The clinical condition, setting, individual/patient-specific characteristics, anticipated duration of wear and desired parameters can inform device selection.

RPM and wearables have been studied in non-COPD respiratory diseases such as in pediatric asthma and during acute viral illness. Depending on the age of the pediatric patient, the forced maneuvers and coordination required for conventional spirometry are difficult for children to complete reproducibly (36). A study by Lundblad et al. (37) demonstrated that respiratory resistance measured during normal (tidal) breathing using a novel handheld portable oscillometer device correlated closely with estimates obtained by conventional oscillometry in children with asthma. User experience questionnaires were favorable, including perceptions by children and their parents that the test was ‘easy’ and that they ‘would use it at home if recommended by their health care provider’, supporting the feasibility of remote lung function monitoring even in children with asthma.

## Remote patient monitoring and wearables in COPD

The development of RPM solutions specifically for patients with COPD is a very active field of clinical research and one that holds great promise. An important foundational 2012 study in this modern field by Yanez et al. (38) utilized existing domiciliary oxygen therapy equipment to detect subtle changes in RR preceding exacerbation. In more than two-thirds of detected exacerbations, the average RR was found to increase as early as 5 days prior to hospitalization and RR closer to hospitalization was increasingly accurate and specific as a predictive ‘biomarker’. In 2015, Borel et al. (39) again leveraged existing patient equipment by analyzing COPD non-invasive ventilation (NIV) recipient data RR and percentage of respiratory cycles triggered (%Trigg) > 75th percentile for two or more of the preceding 5 days were associated with an increased risk of subsequent exacerbation. Daily NIV adherence variations (either > 75th or < 25th percentile) were also linked to subsequent exacerbations (39). This important ‘early’ work laid the foundation for subsequent studies on physiology-based RPM strategies in the ambulatory COPD outpatient population.

A 2017 study by Rubio et al. (40) transported RR into the realm of ‘wearables’ by demonstrating that wearable accelerometers and chest bands provide accurate measures of RR comparable to a gold standard. These were sensitive enough to detect a decrease in RR after an exacerbation, and likewise an increase in RR before a future exacerbation in ambulatory outpatients with COPD (40). To determine whether accurate data collection and good COPD-specific adherence with wearable biometric smartwatches were possible, Wu et al. (41) used quantitative and qualitative methods to demonstrate the feasibility and willingness of participants with COPD to wear smartwatches that collect physiological data. Participants reported desiring active engagement and feedback on their activity, HR, and COPD management.

Walker et al. (3) hypothesized in a 2018 study that remote monitoring of lung function using daily oscillometry measurements was not only possible but moreover would reduce the time to first hospitalization, reduce healthcare costs, and increase quality of life in older patients with COPD and prevalent comorbidities. An advantage of oscillometry, when compared to conventional spirometry, is that patients with COPD can perform this test autonomously and reliably at home without the need for a respiratory therapist (36). Mechanical properties of the lungs during tidal breathing were collected and transmitted remotely (3). While the time to first hospitalization, EQ-5D utility score and quality-adjusted life years (QALYs) were not different between intervention and control groups, the per-patient cost was lower for all subgroups of the intervention group except for those with severe/very severe COPD (3). While potentially underpowered by a low number of events (hospitalizations), these hypothesis-generating secondary outcomes in addition to having demonstrated the acceptability, tolerability, and practicality of remote lung monitoring using oscillometry in older patients with COPD with cardiac comorbidities contributed to the field of RPM in more advanced forms of COPD (3).

Hawthorne et al. (28) investigated the usability and acceptability of a sophisticated biometric wearable vest in patients with COPD both in stable and in acute (peri-exacerbation) conditions. This 2022 study found that while most participants experienced no vest-associated discomfort, a subset of peri-/post-exacerbation participants expressed occasional feelings of restriction and breathlessness thereby influencing their acceptance of the vest (28). The conventional ‘trade-off’ between capturing artifact-free data, versus the patient discomfort associated with some wearable thoracic bands, shirts, and vests, was well-demonstrated in this study. PPG-derived parameters from wearable devices in other COPD-specific studies have demonstrated a reassuringly close correlation between RR and HR with gold-standard acquired measurements (40, 42).

In the same year, Park et al. (43) investigated the feasibility of HR monitoring in COPD using a chest-worn biosensor. As previously reported, this study concluded that HRV was in fact reduced in COPD. Interestingly, this variation was independent of the severity of airflow obstruction, however a correlation between lower HRV and poorer overall health or functional status was observed. Finally, a notable strong variation signal was observed in long-acting inhaled bronchodilator (β-agonist and muscarinic-antagonist) users given the overlapping mechanism of action on the autonomic nervous system, which underscores the sensitivity of these devices and the relevance of accounting for these factors in general in the field of RPM data interpretation.

Most recently, Polsky et al. (44) performed a *retrospective*, non-randomized study on an RPM ‘service’ in patients with COPD which included an undergarment-adhered cardiorespiratory physiologic monitor linked via data capture ‘hub’ to a web-based clinical dashboard. This 2023 study demonstrated that RPM recipients experienced significantly fewer unplanned hospitalizations when compared with usual care. This important study further demonstrates the potential for RPM and wearables to assist in the early detection of ECOPDs. This technology also has the potential to be leveraged towards a better understanding of the physiology (and pathophysiology) of ECOPDs in ambulatory outpatients with COPD, which might help inform future *prospective* large-scale randomized clinical trials evaluating wearable-based RPM interventions in the COPD patient population.

## Emerging artificial intelligence and machine learning applications

Artificial intelligence (AI) has been described as a computer framework which displays ‘human-like intelligence’, whereas machine learning (ML) is a subset of AI that uses statistical models to ‘learn’ from data for designated tasks (45, 46). These methodologies can be leveraged to process, categorize, and analyze substantial amounts of data (45), with a performance which can be autonomously optimized. These properties render AI/ML methodologies ideal for processing substantial physiological datasets, and in keeping with this, the more recent RPM literature has increasingly gravitated towards AI/ML incorporation. In a recent non-COPD study by Grzesiak et al. (24), the capacity of ML models using data obtained from healthy participants who were inoculated with respiratory viruses and wearing a non-invasive ‘wearable’ wristband could accurately predict viral infection status and severity even before symptom onset using ML models. Binary and multiclass random forest classification models, each addressing a distinct time period following inoculation or adopting different criteria to distinguish between ‘infected’ and ‘non-infected’ subjects, were developed. Remarkably, by combining near-continuous wearable-obtained physiologic data with robust ML modelling, it was possible to predict the subsequent severity (mild vs. moderate) of infection at a timepoint which preceded symptom onset by 24 h (24).

Pertaining specifically to the COPD RPM literature, Shah et al. (47) collected data from a large cohort of 110 individuals with COPD over the course of 1 year in order to assess the feasibility of developing a COPD digital health system. A Bluetooth pulse oximeter was paired with a comprehensive questionnaire which obtained near-daily symptom scores and medication use. A finite-state machine learning approach was used to process this extensive dataset, and the model developed was able to effectively classify the health condition of study participants. This study also found that, amongst the parameters collected (HR, RR, and SpO_2_), that SpO_2_ emerged as the most useful in predicting exacerbations.

The applications of AI/ML methodologies towards the development of effective COPD remote monitoring platforms are gaining attention. A recent review (48) details the superior approach of combining AI/ML with remotely acquired data when compared to pre-existing models, platforms and algorithms. Included in this review, Orchard et al. (49), Wu et al. (50) and Fernandez-Granero et al. (51) all place a particular emphasis on the applicability of ML-based approaches in producing sophisticated platforms capable of detecting the very early onset of exacerbations. This predictive capacity, once launched in a real-world clinical setting, could have a substantial impact on disease management in COPD.

## Remote patient monitoring and wearables in COPD: additional applications

Beyond daily outpatient monitoring and ECOPD detection, innovations in RPM and wearable technology can also support and complement longstanding evidence-based standard-of-care interventions in COPD including *pulmonary rehabilitation (PR)* delivery and in reinforcing *self-management behaviors*. Advances in technology now permit the possibility of near-completely ‘remote’ PR delivery, which was particularly important during the COVID-19 pandemic when most in-person PR programs were closed (52). Home-based PR for COPD has been shown to be as effective as center-based PR in improving functional exercise capacity and quality of life (53), and patients with chronic respiratory disease achieved similar effectiveness and safety outcomes to center-based PR as they did with telerehabilitation (53). COPD telerehabilitation may even be able to increase and maintain the persisting benefits of PR (54).

Remote patient monitoring technology can reinforce and support COPD-specific self-management adoption. Real-time feedback on activities, behaviors and physiological changes can make patients with COPD more involved and engaged in their own care and more aware of their condition (40, 55). Patients with COPD have described that this data would empower them by allowing them to link how they feel, a ‘subjective’ experience, to an ‘objective’ measurement such as real-time vital sign information. This may increase awareness, and in some instances, can reassure them about the status of their condition at any given time (55).

## Practical considerations in COPD

While the literature to date on device-based RPM solutions in COPD are encouraging, the ‘real-world’ clinical launch and operation of these platforms remain associated with sizeable challenges. These need to be considered at the earliest stages of platform development/validation (i.e., during the ‘clinical research’ phase) to ensure that the downstream COPD target subpopulations and intended clinical purposes of the platform are maintained. Firstly, the RPM platform device(s) would need to effectively measure the main outcome of interest. Second, the intended patient population and the setting of data collection should be considered (32). COPD-specific factors include patient age (56) and technology ‘literacy’, which might favor the use of simpler devices and interfaces. Generational divides in comfort with technology (software and hardware) may adversely affect patient access to RPM platforms in a notable proportion of the COPD population. Beyond age and generation, individual educational background, professional work experience, and personal experience with technology are relevant (57). Moreover, because the goal of RPM is real-time near-continuous data collection, the devices should be as comfortable as possible for prolonged wear. Finally, an extensive battery life, intuitive or even automatic data upload processes, and convenient platform access by the treating clinical team must all be factored into the design of wearable device-based RPM platforms intended for ‘real-world’ clinical use.

## Ethical considerations in COPD

Beyond the practical issues in developing, testing, and launching device-based RPM platforms in the COPD patient population, there are also ethical principles which are paramount. Firstly, while it might be challenging for the ‘highest risk’ patients or those with the most advanced forms of COPD to participate in clinical research studies, researchers must find ways to include these patients in particular given that RPM strategies are most likely to be useful and cost-effective in this clinical subpopulation as it relates to reducing patient and health system burden. For example, if the platform intends to detect new exacerbations in those patients at highest risk, then this high-risk subpopulation (rather than, for example, patients with milder disease or infrequent exacerbations) must be enrolled and studied in these trials. Likewise, the intention to serve traditionally vulnerable and marginalized populations through these technological advances (58) must be met with a purposeful and equitable commitment of the clinical investigator to include these patients in RPM clinical research studies, to minimize the risk of their subsequent exclusion at the time of downstream clinical launch. Finally, an ongoing and organized strategy at hospital administrative, governmental, and even international levels in order to oversee and regulate ethical aspects and best practices in the utilization of RPM technologies and patient data is critical. The priority must always be the patient, their well-being, their right to confidentiality and privacy, and their right to autonomy. The highly sensitive data that can be collected by these platforms must be protected, anonymized, and safely stored.

## Conclusion: current landscape and future directions

Although significant progress has been made, there remains a need to continually develop and refine existing versions of device-based RPM platforms such that ever-improving sensors, longer battery lives, smaller sizes, more efficient and precise computational algorithms, and enhanced data security features can be harnessed to effectively face the many challenges in the modern care of COPD (15). Research dedicated to the bottom-line clinical efficacy of these platforms in COPD in a prospective manner is necessary before more widespread adoption in the clinical sphere can occur. The future interaction between wearable biometric devices, sophisticated platforms, and harnessing the power of ‘big data’ and AI/ML methods (48–51) makes this an exciting and promising field which will no doubt shape the future of healthcare.

## Author contributions

F-AC, OI, and BR developed the manuscript template, performed the literature review, finalized the content and relevant themes, and wrote the manuscript. All authors have given agreement to be accountable for all aspects of the work in ensuring that questions related to the accuracy or integrity of any part of the article are appropriately investigated and resolved.

## Funding

Publication-associated fees supported by the McGill University Health Centre (MUHC) Department of Medicine Contract Academic Staff (CAS) Research Award.

## Conflict of interest

BR reports the following Conflicts of Interest: *Honoraria* from the Canadian Thoracic Society (CTS—presenter and educational content creator), CHEST (educational content creator), Respiplus non-profit (educational content creator), Alberta Kinesiology Association (AKA—educational webinar presenter), Association des Pneumologues de la Province du Québec (APPQ—presenter), McGill University Continuing Professional Development (CPD—presenter) and from GSK (educational session moderator); *Research funding* as Principal Investigator from the McGill University Health Centre (MUHC) Department of Medicine Contract Academic Staff (CAS) Research Award, the Quebec Respiratory Health Network (QHRN), the *Ministère de l’Éducation et le Ministère de l’Enseignement Supérieur* Innovation and Partnership Program (McGill University and Thorasys, Inc.), and the MUHC Foundation/MCI Respiratory Research Campaign Innovation Grant.

The remaining authors declare that the research was conducted in the absence of any commercial or financial relationships that could be construed as a potential conflict of interest.

## Publisher’s note

All claims expressed in this article are solely those of the authors and do not necessarily represent those of their affiliated organizations, or those of the publisher, the editors and the reviewers. Any product that may be evaluated in this article, or claim that may be made by its manufacturer, is not guaranteed or endorsed by the publisher.
