# Supplementary material for: Remote patient monitoring strategies and wearable technology in chronic obstructive pulmonary disease
Source: Front Med (Lausanne). 2023 Aug 17;10:1236598. doi: 10.3389/fmed.2023.1236598 (PMC10470466; doi:10.3389/fmed.2023.1236598)
Supplement: Supplementary file 1 [file Table_1.docx]

Supplementary Material

Remote patient monitoring strategies and wearable technology in chronic obstructive pulmonary disease

Felix-Antoine Coutu^1,4^, Olivia C. Iorio^1^, Bryan A. Ross^1,2,3,4*^

^1^Respiratory Epidemiology and Clinical Research Unit, Centre for Outcomes Research and Evaluation, Research Institute of the McGill University Health Centre, Montreal, Quebec, Canada

^2^Division of Respiratory Medicine, Department of Medicine, McGill University Health Centre, Montreal, Quebec, Canada

^3^Montreal Chest Institute, McGill University Health Centre, Montréal, Québec, Canada

^4^McGill University, Montréal, Québec, Canada

***Correspondence:**Dr. Bryan A. Ross

Respiratory Epidemiology and Clinical Research Unit, Research Institute of the McGill University Health Centre

5252 De Maisonneuve, room 3D.57, Montreal, QC, H4A 3S5, Canada.

E-mail: [bryan.ross@mcgill.ca](mailto:bryan.ross@mcgill.ca).

***Focused Summary of Remote Patient Monitoring in COPD: Methodology***

In order to summarize the topic of “remote patient monitoring and wearable technology in COPD” succinctly and clearly for this Mini Review, a semi-structured approach was followed for the background literature review and drafting of the manuscript. Firstly, the authors (FAC, OCI, and BAR) co-developed principal themes and sub-themes which were to be included. In order to select and include the most relevant articles, the appropriate search strategies were developed by choosing representative search terms for each theme and sub-theme. These also provided a structure for the subsequent manuscript, as ‘themes’ and ‘sub-themes’ evolved into the ‘sections’ and ‘sub-sections’ of the Discussion section.

The main search strategy used was the following: *((((("exacerbation of COPD") OR ("COPD exacerbation")) OR ("AECOPD")) OR ("ECOPD")) AND (("predict*") OR ("identification"))) AND ((((("remote") OR ("home")) OR ("telemonitoring")) OR ("telehealth")) OR ("wearable*"))*. This initial search yielded 105 results. Each of these articles was independently reviewed and evaluated by each author. Authors were permitted to retrieve, review, and put forth additional articles cited from the original 105 articles if they were deemed to be relevant to the Mini Review themes and sub-themes. Following this independent review and appraisal process, all authors met to discuss which articles were most relevant to the co-developed themes and sub-themes identified. The list of articles to be presented in the Mini Review was finalized after consensus was reached by all authors.

Given the expanding role of artificial intelligence (AI) and machine learning (ML) in this field, particularly in the more recent published literature, a separate complementary search was performed to identify relevant AI/ML-related remote patient monitoring studies using the following strategy: *((("Chronic obstructive pulmonary disease") OR ("COPD")) AND (("Machine learning") OR ("Artificial intelligence"))) AND ((("Management") OR ("Monitoring")) OR ("Telemonitoring")).* Review articles covering AI/ML and remote patient monitoring were also sought, which yielded a thorough scoping review (De Ramón Fernández *et al*. (2021)). Articles which focused on exacerbation prediction from telemonitoring-acquired physiological data (Fernandez-Granero *et al.* (2018), Orchard *et al*. (2018), and Wu *et al*. (2021)) were extracted for inclusion into the mini-review.

**Table 1.** Description and summary of featured studies

| **Author** | **Equipment & Parameters** | **Results & Field Relevance** |
| --- | --- | --- |
| ***Non-Portable Devices*** | | |
| **Yañez *et al.*** (2012) (1) | RR monitor on home oxygen supply system (*RR*) | In 70% of patients that experienced an ECOPD, RR increased during the 5 days preceding hospitalization. The increase was approximately 4.4 breaths/minute (30%) and 2.3 breaths/minute (15%) at 24h and 48h prior to hospitalization, respectively.  Demonstrated the feasibility of RR remote monitoring in patients with COPD receiving home oxygen, as well as the possibility to predict ECOPD using RR changes. |
| **Type of Study:** Prospective observational |  |  |
| **Sample Size:** 89 |  |  |
| **Population:** COPD with long-term home oxygen |  |  |
| **Follow-up duration:** 3 months |  |  |
| **Objectives**  Determine whether the increase in RR preceding episodes of ECOPD can be detected days before hospitalization. |  |  |
| **Borel *et al.*** (2015) (2) | Home NIV system (*RR, %Trigg and NIV daily use*) | Risk of ECOPD was increased when RR and %Trigg were >75^th^ percentile on ≥ 2 days out of 5. High (>75^th^ percentile) or low (<25^th^ percentile) daily use of NIV was also associated with an increased exacerbation risk.  Demonstrated that ECOPD can be detected earlier through the remote measurement of respiratory parameters. |
| **Type of Study:** Prospective observational |  |  |
| **Sample Size:** 64 |  |  |
| **Population:** COPD on home NIV |  |  |
| **Follow-up duration:** 6 months |  |  |
| **Objectives**  Determine whether daily variations in three parameters recorded by NIV software predict the risk of exacerbation in patients with COPD treated by home NIV. |  |  |
| ***Oscillometry*** | | |
| **Walker *et al.* (2018)** (3) | Oscillometer *(respiratory mechanical impedance, inspiratory resistance, inspiratory reactance, the difference between inspiratory and expiratory reactance, and an index of the presence of tidal expiratory flow limitation)*  Wrist-worn portable device *(BP, SpO_2_, HR, body T°)* | No difference between intervention and control group in the rate of moderate exacerbations, hospitalizations, or the number of patients free from hospital admission.  Reduction in mean cost per patient in the intervention group compared to the control group.  No difference between groups in the TTFH and EQ-5D utility scores.  53% reduction in hospitalization rate for those hospitalized for COPD exacerbation in the intervention group, and significant difference in the mean duration of hospital stay and the total days of hospitalization after an emergency admission.  Demonstrated the clinical feasibility and acceptability of remote FOT and cardiac monitoring in the COPD patient population. Suggested that using objectively defined criteria for clinical deterioration in at-risk patients with COPD may be useful in preventing hospitalization. |
| **Type of Study:** RCT |  |  |
| **Sample Size:** 312 |  |  |
| **Population:** COPD (GOLD II-IV) |  |  |
| **Follow-up duration:** 9 months |  |  |
| **Objectives**  To evaluate the efficacy of remote monitoring by monitoring respiratory (oscillometry) and cardiac parameters. |  |  |
| **Lundblad *et al.* (2021)** (4) | REOM device and conventional oscillometer (*Airway opening peak pressure and Resistance at exhalation-occlusion ratio*) | The resistance measured on the Tremoflo at 5Hz and 19Hz was most comparable to the measurements at post-peak flow and peak flow, respectively.  The resistance of the healthy controls was significantly different from the controlled asthmatics, and uncontrolled asthmatics.  The measurements at peak flow and post-peak flow on the REOM were strongly correlated with the Tremoflo at 5Hz and 19Hz.  No statistically significant difference between conventional oscillometry at 5Hz and 19Hz and measurements at post-peak flow and peak flow on the REOM, respectively.  The measurements at post-peak flow and peak flow on the REOM and 5Hz and 19Hz on expiration on conventional oscillometry, respectively, were strongly correlated.  Demonstrated the practicality and future potential of remotely monitoring lung mechanical properties with a handheld portable device, even amongst pediatric patients with asthma. |
| **Type of Study:** Cross-sectional |  |  |
| **Sample Size:** 30 |  |  |
| **Population:** Healthy controls, ‘controlled’ asthma, and ‘poorly controlled’ asthma |  |  |
| **Follow-up duration:** One session |  |  |
| **Objectives**  1°: Compare the resistance obtained by the flow interrupter technique on the REOM with the resistance obtained by conventional oscillometry.  2°: Determine the correlation and agreement between the interrupter resistance parameters with the oscillometry parameters that reflect whole lung versus central airway resistance. |  |  |
| ***Portable Devices*** | | |
| **Shah *et al.* (2017)** (5) | Pulse oximeter (*SpO_2_, pulse rate and RR (via PPG)*) | Demonstrated and quantified the predictive power of 3 vital signs. SpO_2_ was the most predictive, followed by RR, and pulse rate was the least predictive.  Demonstrated reliable parameter acquisition through home monitoring using only a pulse oximeter.  Presented a systematic approach to modeling COPD exacerbation based on finite-state machines (FSM) that provides a deeper insight into how a COPD progresses over time.  Remotely collected vital signs can be used for ECOPD analysis and detection as well as to enhance symptom worsening comprehension. |
| **Type of Study:** RCT |  |  |
| **Sample Size:** 110 |  |  |
| **Population:** Stable COPD |  |  |
| **Follow-up duration:** 12 months |  |  |
| **Objectives**  1°: Develop a systematic and reproducible approach to exacerbation identification and to track the progression of patient condition during remote monitoring.  2°: Develop a robust algorithm able to predict ECOPD, based on vital signs acquired from a pulse oximeter. |  |  |
| **L’Her *et al.* (2019)** (6) | Retrospective Evaluation: ECG, SYNAPSE software (*RR via PPG*)  Prospective Evaluation: Pulse oximeter (*RR (via PPG))* | RR derived from PPG waveform is comparable to both reference measures (manual and ventilator RR), with low bias and deviation.  The correlation was similar for mechanically ventilated versus spontaneously breathing.  Modestly lower correlation was observed in patients with atrial fibrillation.  Demonstrated that a PPG waveform algorithm can be used to accurately measure RR when implemented in a biomedical device, enabling concurrent monitoring of both RR and SpO_2_ through a portable pulse oximeter. |
| **Type of Study:** Observational (retrospective ‘evaluation’ phase, prospective ‘estimation’ phase) |  |  |
| **Sample Size:** 201 |  |  |
| **Population:** Critically ill patients with various severe conditions who have been admitted to the ICU, with the majority having respiratory failure. |  |  |
| **Follow-up duration:** Not disclosed. |  |  |
| **Objectives**  1°: To evaluate the accuracy of RR measurements using a reflection-mode PPG signal analysis in pathological conditions.  2°: To validate PPG-derived RR implementation within medical devices. |  |  |
| ***Wearables*** | | |
| **Rubio *et al.* (2017)** (7) | Chest-band (*HR, R-R interval, RR, ECG, posture, and physical activity level*) | Mean RR at rest decreased progressively following an exacerbation.  A large inter-individual variation was observed between patients in terms of RR. Some patients did not demonstrate RR changes in the recovery period.  One patient had a second exacerbation during monitoring and demonstrated an increase in RR and HR paired with the occurrence of symptoms, which anecdotally confirmed the ability of the device to capture pre-ECOPD physiologic changes.  Demonstrated that wearable devices can accurately measure RR remotely for prolonged periods of time and are sensitive enough to detect the changes associated with ECOPDs. |
| **Type of Study:** Prospective observational |  |  |
| **Sample Size:** 18 |  |  |
| **Population:** Post-ECOPD |  |  |
| **Follow-up duration:** 3-6 weeks |  |  |
| **Objectives**  1°: To examine the ability of the best-performing device in detecting any changes in RR.  2°: If the primary outcome proved feasible, to assess those at risk of future exacerbation by investigating whether RR changes can identify exacerbations at an early stage. |  |  |
| **Hawthorne *et al.* (2022)** (8) | Biometric vest (*RR, HR, skin T°, physical activity*) | Confirmed that biometric vests can be used in patients with COPD and have the potential to monitor vital signs both post-hospitalization for acute exacerbation and throughout the stable (non-exacerbation) phase.  Data quality was found to be influenced by body composition/shape. The exacerbation subgroup spent significantly less time ambulatory, and were observed to have a lower HR signal quality.  Qualitative results suggested that some patients felt that the vest was restrictive and made them feel breathless.  Some patients had to be excluded because no commercially available vest size could fit their chest size.  The exacerbation subgroup was less adherent to vest wear than the chronic COPD subgroup, with more observed withdrawals in that subgroup).  Demonstrated the ability to continuously measure vital signs using a biometric vest in individuals with COPD. However, the study also highlighted specific challenges in the post-ECOPD setting, creating a dilemma between obtaining artifact-free data and minimizing patient discomfort associated with certain wearables. |
| **Type of Study:** Prospective observational |  |  |
| **Sample Size:** 100 |  |  |
| **Population:** Post-ECOPD and stable COPD |  |  |
| **Follow-up duration:** 6 weeks |  |  |
| **Objectives**  1°: Feasibility, usability, and acceptability of continuous vital sign monitoring using a wearable device after hospitalization for acute exacerbation of COPD, and during the stable (non-exacerbation) phase.  2°: Investigate patient characteristics associated with data quality and adherence, and to determine differences in feasibility between stable COPD and post-exacerbation participants. |  |  |
| **Park *et al.* (2022)** (9) | Chest strap (*R-R interval data and HRV*) | HRV is reduced in patients with COPD, but this reduction is independent of the severity of airflow obstruction.  HRV had a weak to moderate correlation with functional status/health in participants with COPD. Lower SDNN is correlated with poor health/functional status.  A strong correlation was observed between HRV (measured by the wearable biosensor) and use of inhaled bronchodilators (β-agonist and muscarinic-antagonist), due to effects on the autonomic nervous system.  Demonstrated that wearable chest bands can measure HRV in patients with COPD regardless of severity. The results regarding the impact of inhalers on HRV emphasize the importance for researchers and clinicians to carefully consider these types of effects when interpreting remote monitoring data. |
| **Type of Study:** Cross-sectional |  |  |
| **Sample Size:** 79 |  |  |
| **Population:** Stable COPD |  |  |
| **Follow-up duration:** One session |  |  |
| **Objectives**  1°: Demonstrate the feasibility of monitoring HRV using a chest-worn wearable biosensor.  2°: Extract data from a clinical study of patients with moderate to severe COPD to determine whether there is an association between HRV and other correlates of functional status and health. |  |  |
| **Polsky *et al.* (2023)** (10) | Undergarment waistband- adhering cardiorespiratory sensors (*HR, RR, continuous respiratory force, activity, duration worn, and step count*) | The number of all-cause and cardiopulmonary hospitalizations decreased significantly in the 12 months following RPM initiation.  There was a trend in reduced length of stay during all-cause and cardiopulmonary hospitalizations, which did not meet statistical significance.  Number of outpatient clinic office visits increased significantly following RPM initiation.  These retrospective results support the effectiveness of RPM in sustained/long-term outpatient COPD management and suggests that a reduction in healthcare costs and burden is possible by shifting care from the more resource-intensive inpatient hospitalization setting to the outpatient ambulatory clinic setting. |
| **Type of Study:** Retrospective observational |  |  |
| **Sample Size:** 126 |  |  |
| **Population:** COPD |  |  |
| **Follow-up duration:** 12 months (pre-) and 12 months (post-) RPM initiation |  |  |
| **Objectives**  To determine whether an RPM ‘system’ coupled to a protocol intended for timely medical intervention can improve healthcare resource utilization. |  |  |

| ***Machine Learning and Artificial Intelligence*** | | |
| --- | --- | --- |
| **Orchard *et al.* (2018)** (11) | Portable oximeter (*HR, SpO_2_*)  Tablet (*daily symptom score, medication*)  UK Met Office Healthy Outlook (*data on season, humidity, temperature, air quality, and rates of influenza-like illness*) | ML algorithms significantly improved accuracy, reducing false alerts by half compared to the traditional method of telemonitoring data analysis.  The algorithm accurately categorized patients by admission risk and predicted the decision to start corticosteroids within 24h.  Meteorological data had a minimal impact on the model's predictive accuracy.  These results support the application of AI/ML-based methods to analyze symptom and vital sign data from telemonitoring platforms given that predictive performance surpassed that of ‘conventional’ predictive algorithms. |
| **Type of Study:** Data extracted from an RCT |  |  |
| **Sample Size:** 146 |  |  |
| **Population:** Moderate to severe COPD |  |  |
| **Follow-up duration:** 16 months |  |  |
| **Objectives**  1°: To determine whether ML techniques applied to telemonitoring datasets improve prediction of hospital admissions and decisions to start corticosteroids  2°: To determine whether the addition of weather data further enhances this predictive capacity. |  |  |
| **Fernandez-Granero *et al.* (2018)** (12) | Respiratory sensor (*respiratory sound frequencies*) | The system demonstrated the ability to predict symptom-based episodes with a margin, on average, of 4.4 days before exacerbation onset.  The system showed a high sensitivity (78.1%) and NPV (83.9%), and very high specificity (95.9%) and PPV (94.1%).  These study results support the advantage of AI applications when coupled to RPM platforms in terms of early detection of ECOPDs. |
| **Type of Study:** Prospective observational |  |  |
| **Sample Size:** 16 |  |  |
| **Population:** COPD |  |  |
| **Follow-up duration:** 6 months |  |  |
| **Objectives**  Explore the performance of daily home recordings of respiratory sounds for early detection of symptom-based exacerbations by using computerized analysis and artificial intelligence techniques. |  |  |
| **Wu *et al.* (2021)** (13) | Wristband (*step count, stairs climbed, distance, calorie consumption, HR, and sleep status*)  Home air quality-sensing device (*PM_2.5_, temperature, humidity*) | The model achieved a very high sensitivity (94%) and specificity (90.4%) for 7-day ECOPD prediction.  The most ‘important’ variables in the model were daily steps walked, stairs climbed, and daily distance moved.  ECOPD prediction model performance was found to be substantially improved by also adding objective lifestyle and environmental data.  Wearable and home air quality devices, along with prediction algorithms, when paired together, are highly accurate in forecasting ECOPD risk. |
| **Type of Study:** Prospective observational |  |  |
| **Sample Size:** 67 |  |  |
| **Population:** COPD |  |  |
| **Follow-up duration:** 4 months |  |  |
| **Objectives**  To develop a prediction system using lifestyle data, environmental factors, and patient symptoms for the early detection of ECOPDs in the upcoming 7 days. |  |  |
| **Grzesiak *et al.* (2021)** (14) | Wristband (*HR, skin T°, electrodermal activity, and movement*) | ML models developed from wristband wearable-derived data was able topredict infection status and infection severity 12h to 36h before symptom onset.  Most of these models reached >80% accuracy.  The majority of physiological changes in response to viral exposure, which can be used to predict the severity of future illness, were observed to occur within 12h to 24h of inoculation.  This study effectively demonstrated that near-continuous remotely collected data obtained from healthy participants inoculated with common respiratory illnesses can be combined with ML modeling in order to accurately predict infection severity 24h before the onset of symptoms. |
| **Type of Study:** Prospective cohort |  |  |
| **Sample Size:** 49 (H1N1:31; rhinovirus:18) |  |  |
| **Population:** Healthy subjects (inoculated in protocol) |  |  |
| **Follow-up duration:** H1N1: 11 days, rhinovirus: 9 days |  |  |
| **Objectives**  To evaluate the feasibility of using data obtained from a non-invasive, wrist-worn wearable biometric monitoring sensor to detect pre-symptomatic viral infection after exposure and to predict infection severity in participants inoculated with H1N1 influenza or human rhinovirus. |  |  |
| **De Ramòn Fernández *et al.* (2021)** (15) | Not applicable (*most studies collected vital sign data, symptom data, and demographic/risk factor information*). | AI/ML methodologies are increasingly being used in developing predictive modeling in COPD management platforms.  The principal emerging objectives are to identify and classify COPD (in the ‘stable’ chronic disease state), to predict and detect new exacerbations, to determine worsening of the chronic condition, and to predict death in patients with COPD.  AI/ML platforms are effective and demonstrate excellent performance in terms of accuracy, precision, sensitivity, and specificity results.  AI/ML methodologies appear to effectively aid in diagnosing, classifying, and managing COPD in both the ‘chronic’ and ‘acute’ disease conditions, with improved performance over conventional methods.  AI/ML may have the capacity to enhance COPD management by better diagnosing and classifying chronic disease and by improving early detection of exacerbations. |
| **Type of Study:** Scoping review |  |  |
| **Sample Size:** 67 original research studies (variable sample sizes) |  |  |
| **Population:** COPD |  |  |
| **Follow-up duration:** Not applicable |  |  |
| **Objectives**  To evaluate how AI is being applied in the modern management of COPD, including an analysis of the objectives tested, the algorithms used, and an appraisal of the study results reported. |  |  |

*Abbreviations:* AI: Artificial intelligence; BP: Blood pressure; Body T°: Body temperature; RR: Respiratory Rate; COPD: Chronic obstructive pulmonary disease; ECG: Electrocardiogram; ECOPD: Exacerbation of chronic obstructive pulmonary disease; EQ-5D: EuroQol 5D utility index score; FOT: Forced oscillation technique; HR: Heart rate; HRV: Heart rate variability; ICU: Intensive care unit; ML: Machine learning; ; NIV: non-invasive ventilation; NPV: Negative predictive value;; PM_2.5_: Fine particulate matter; PPG: Photoplethysmography; PPV: Positive predictive value; RCT: Randomized controlled trial; REOM: Rapid Expiratory Occlusion Method; RPM: Remote patient monitoring; RR: Respiratory rate; SDNN: Standard deviation of the mean of all normal R-R intervals; SpO_2_: Saturation of peripheral oxygen; %Trigg: percentage of respiratory cycles triggered by the patient.

**Bibliography**

1. Yañez AM, Guerrero D, Pérez de Alejo R, Garcia-Rio F, Alvarez-Sala JL, Calle-Rubio M, et al. Monitoring breathing rate at home allows early identification of COPD exacerbations. Chest. 2012;142(6):1524-9.

2. Borel JC, Pelletier J, Taleux N, Briault A, Arnol N, Pison C, et al. Parameters recorded by software of non-invasive ventilators predict COPD exacerbation: a proof-of-concept study. Thorax. 2015;70(3):284-5.

3. Walker PP, Pompilio PP, Zanaboni P, Bergmo TS, Prikk K, Malinovschi A, et al. Telemonitoring in chronic obstructive pulmonary disease (CHROMED). A randomized clinical trial. Am J Respir Crit Care Med. 2018;198(5):620-8.

4. Lundblad LKA, Blouin N, Grudin O, Grudina L, Drapeau G, Restrepo N, et al. Comparing lung oscillometry with a novel, portable flow interrupter device to measure lung mechanics. J Appl Physiol 2021;130(4):933-40.

5. Shah SA, Velardo C, Farmer A, Tarassenko L. Exacerbations in Chronic Obstructive Pulmonary Disease: Identification and Prediction Using a Digital Health System. J Med Internet Res. 2017;19(3):e69.

6. L’Her E, N’Guyen Q-T, Pateau V, Bodenes L, Lellouche F. Photoplethysmographic determination of the respiratory rate in acutely ill patients: validation of a new algorithm and implementation into a biomedical device. Ann Intensive Care. 2019;9(1):11.

7. Rubio N, Parker RA, Drost EM, Pinnock H, Weir CJ, Hanley J, et al. Home monitoring of breathing rate in people with chronic obstructive pulmonary disease: observational study of feasibility, acceptability, and change after exacerbation. Int J Chron Obstruct Pulmon Dis. 2017;12:1221-31.

8. Hawthorne G, Greening N, Esliger D, Briggs-Price S, Richardson M, Chaplin E, et al. Usability of wearable multiparameter technology to continuously monitor free-living vital signs in people living with chronic obstructive pulmonary disease: prospective observational study. JMIR Hum Factors. 2022;9(1):e30091.

9. Park S-C, Saiphoklang N, Jung D, Gomez D, Phillips JE, Dolezal BA, et al. Use of a wearable biosensor to study heart rate variability in chronic obstructive pulmonary disease and its relationship to disease severity. Sensors. 2022;22(6):2264.

10. Polsky M, Moraveji N, Hendricks A, Teresi RK, Murray R, Maselli DJ. Use of remote cardiorespiratory monitoring is associated with a reduction in hospitalizations for subjects with COPD. Int J Chron Obstruct Pulmon Dis. 2023;18:219-29.

11. Orchard P, Agakova A, Pinnock H, Burton CD, Sarran C, Agakov F, et al. Improving prediction of risk of hospital admission in chronic obstructive pulmonary disease: application of machine learning to telemonitoring data. J Med Internet Res. 2018;20(9):e263.

12. Fernandez-Granero MA, Sanchez-Morillo D, Leon-Jimenez A. An artificial intelligence approach to early predict symptom-based exacerbations of COPD. Biotechnol Biotechnol Equip. 2018;32(3):778-84.

13. Wu CT, Li GH, Huang CT, Cheng YC, Chen CH, Chien JY, et al. Acute exacerbation of a chronic obstructive pulmonary disease prediction system using wearable device data, machine learning, and deep learning: development and cohort study. JMIR Mhealth Uhealth. 2021;9(5):e22591.

14. Grzesiak E, Bent B, McClain MT, Woods CW, Tsalik EL, Nicholson BP, et al. Assessment of the feasibility of using noninvasive wearable biometric monitoring sensors to detect Influenza and the common cold before symptom onset. JAMA Netw Open. 2021;4(9):e2128534.

15. De Ramón Fernández A, Ruiz Fernández D, Gilart Iglesias V, Marcos Jorquera D. Analyzing the use of artificial intelligence for the management of chronic obstructive pulmonary disease (COPD). Int J Med Inform. 2021;158:104640.
